# Categorization and Characterization of Time Domain CMOS Temperature Sensors

**DOI:** 10.3390/s20226700

**Published:** 2020-11-23

**Authors:** Sangjin Byun

**Affiliations:** Division of Electronics and Electrical Engineering, Dongguk University, Seoul 04620, Korea; sjbyun@dongguk.edu; Tel.: +82-2-2260-3331

**Keywords:** temperature sensor, time domain, frequency, period, delay time, temperature estimation function, categorization, characterization, CMOS integrated circuits

## Abstract

Time domain complementary metal-oxide-semiconductor (CMOS) temperature sensors estimate the temperature of a sensory device by measuring the frequency, period and/or delay time instead of the voltage and/or current signals that have been traditionally measured for a long time. In this paper, the time domain CMOS temperature sensors are categorized into twelve types by using the temperature estimation function which is newly defined as the ratio of two measured time domain signals. The categorized time domain CMOS temperature sensors, which have been published in literature, show different characteristics respectively in terms of temperature conversion rate, die area, process variation compensation, temperature error, power supply voltage sensitivity and so on. Based on their characteristics, we can choose the most appropriate one from twelve types to satisfy a given specification.

## 1. Introduction

Temperature sensors have been widely used for thermal monitoring in various applications such as military, aerospace, scientific research, industry, agriculture, medicine, transportation and so on. Specifically, the thermal management of processors and memories, the ambient temperature monitoring for smart greenhouses, the human body temperature tracking for medical purposes and so on are the representative examples of temperature sensor applications.

The most popular temperature sensors used today are the thermocouple, resistive temperature device (RTD), thermistor and integrated silicon-based sensors. Among them, the last one is known to have the relatively narrow measurement range of −55 °C to 150 °C and the relatively low measurement accuracy of ±1 °C compared to the others. However, it is attractive in that it can be easily integrated on the same silicon with the target system, it is not expensive, and it has the relatively fast conversion rate [1].

**Figure 1:**
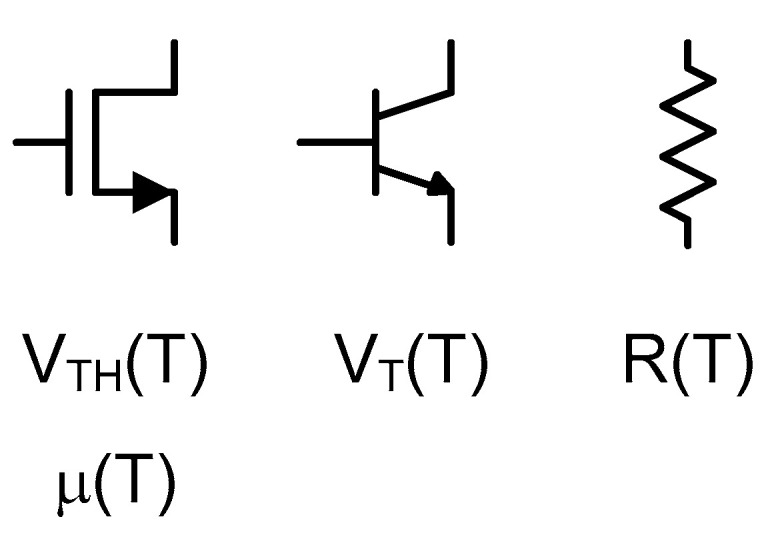
Sensory devices.

Temperature can be estimated by measuring the voltage and/or current signals of a sensory device of which certain characteristic varies depending on the temperature. For integrated circuits, sensory devices mean a complementary metal-oxide-semiconductor (CMOS) transistor, a bipolar junction transistor (BJT) transistor and a kind of passive resistor as shown in Figure 1. For example, a CMOS transistor has the threshold voltage, V_TH_(T), and the mobility, μ(T), which change depending on the temperature and a BJT transistor has the thermal voltage, V_T_(T), which also changes with the temperature. Passive resistors also have the temperature dependent resistance, R(T). So, we can measure the temperature indirectly through the voltage and/or current signals of a sensory device.

Measuring something is to represent it by using a ratio of two quantities. Length is represented by the ratio of the distance between two endpoints and the unit reference like 1 mm or 1 μm and weight is represented by the ratio of the mass and the unit reference like 1 g or 1 mg. However, since temperature cannot be directly measured by the integrated circuits, we should instead measure the temperature dependent voltage and/or current signals and represent it by using the ratio of the measured quantities [2,3,4,5,6,7,8,9,10,11,12,13,14,15,16,17]. This ratio is later converted to the temperature in the unit of °C or ºF.

As the CMOS process scales down and the supply voltage shrinks, the recent trend is to prefer the time domain signals such as the frequency, period and delay time to the voltage and current signals [18,19,20,21,22,23,24,25,26,27,28,29,30,31,32]. So, the time domain CMOS temperature sensors are holding more attraction than the traditional CMOS temperature sensors based on the voltage and/or current signals.

Therefore, this review paper discusses on the categorization and characterization of the time domain CMOS temperature sensors. In Section 2, the temperature estimation function is defined as the ratio of two measured time domain signals and by using the defined temperature estimation function it is shown that all the time domain CMOS temperature sensors can be categorized into twelve types. In Section 3, the temperature estimation functions of the previously published time domain CMOS temperature sensors in literature are derived and by using the derived temperature estimation functions it is shown that they have different characteristics respectively in terms of temperature conversion rate, die area, process variation compensation, temperature error, power supply voltage sensitivity and so on. Finally, the summary and discussion are given in Section 4 and the conclusion is given in Section 5.

## 2. Categorization

**Figure 2:**
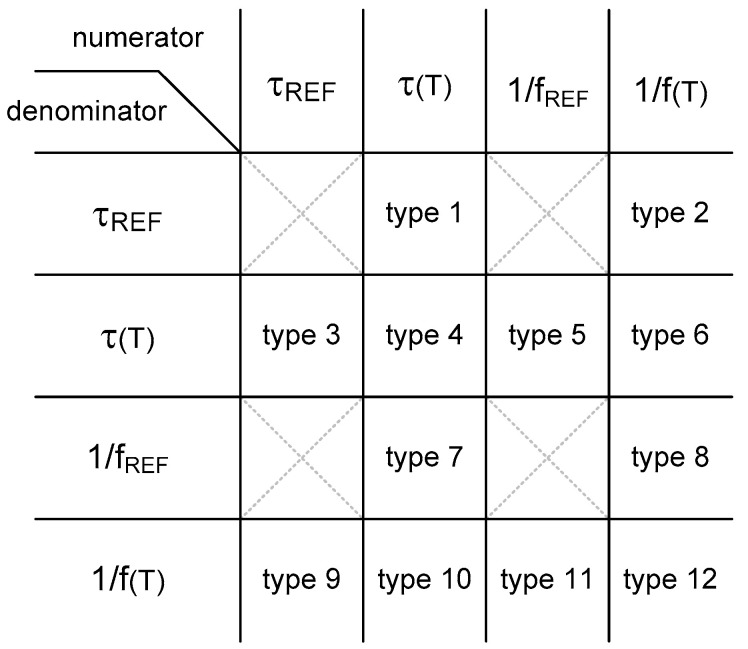
Categorization based on the temperature estimation function.

**Figure 3:**
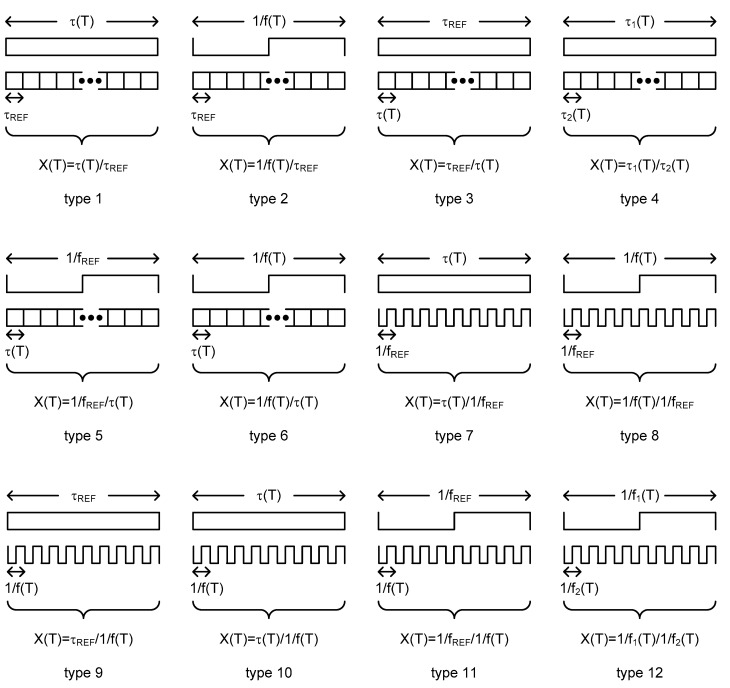
Conceptual diagrams of each type.

To categorize the time domain CMOS temperature sensors, I define the temperature estimation function as the ratio of two measured time domain signals in this paper. The time domain CMOS temperature sensors can be categorized on the basis of the types of the temperature estimation functions. The time domain signals, which are generally used in time domain CMOS temperature sensors, are the frequency, period and delay time. Among them, since the frequency is the reciprocal of the period, we can consider the period and the delay time as the representative time domain signals. The period is related to an oscillator or a clock signal applied externally and the delay time is related to a delay cell or a delay line. If we denote the temperature dependent period and the temperature independent period as 1/f(T) and 1/f_REF_, respectively, the temperature dependent delay time and the temperature independent delay time can also be denoted as τ(T) and τ_REF_, respectively. Therefore, from these 4 kinds of time domain signals, we can obtain overall 16 types of temperature estimation functions as summarized in Figure 2. However, if we exclude the 4 types of temperature estimation functions which are not dependent of the temperature at all, there exist only 12 types of temperature estimation functions. I have named them one by one as type 1, type 2, …, type 12 and drawn their conceptual diagrams for intuitive understanding in Figure 3. Here, X(T) is the temperature estimation function.

**Figure 4:**
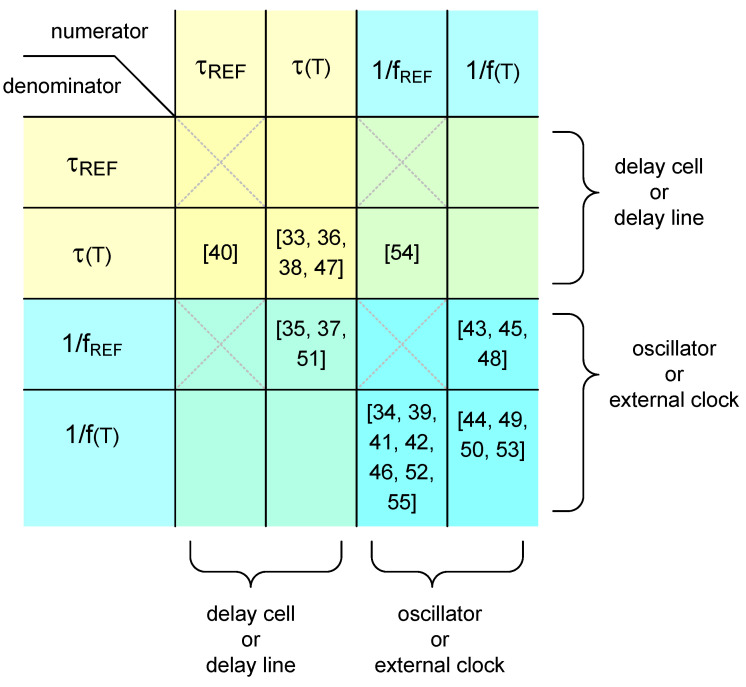
Categorization of the time domain CMOS temperature sensors previously published in literature.

According to my survey, a few tens of papers on time domain CMOS temperature sensors have been published since 2005 [33,34,35,36,37,38,39,40,41,42,43,44,45,46,47,48,49,50,51,52,53,54,55]. They can all be categorized on the basis of their temperature estimation functions. As shown in Figure 4, there are the types which have been adopted preferably and the other types which have never been adopted until now. In the following section, the temperature estimation functions are derived for the previously published time domain CMOS temperature sensors which were categorized into each type. And, by using the derived temperature estimation function, it is shown that the time domain CMOS temperature sensors have different characteristics in terms of temperature conversion rate, die area, process variation compensation, temperature error, power supply voltage sensitivity and so on.

## 3. Characterization

The previously published time domain CMOS temperature sensors in literature [33,34,35,36,37,38,39,40,41,42,43,44,45,46,47,48,49,50,51,52,53,54,55] can be categorized into one of the types 3, 4, 5, 7, 8, 11 and 12, respectively, as shown in Figure 4. In this section, their temperature estimation functions, X(T), are derived first and then characterized as follows.

### 3.1. Type 3

**Figure 5:**
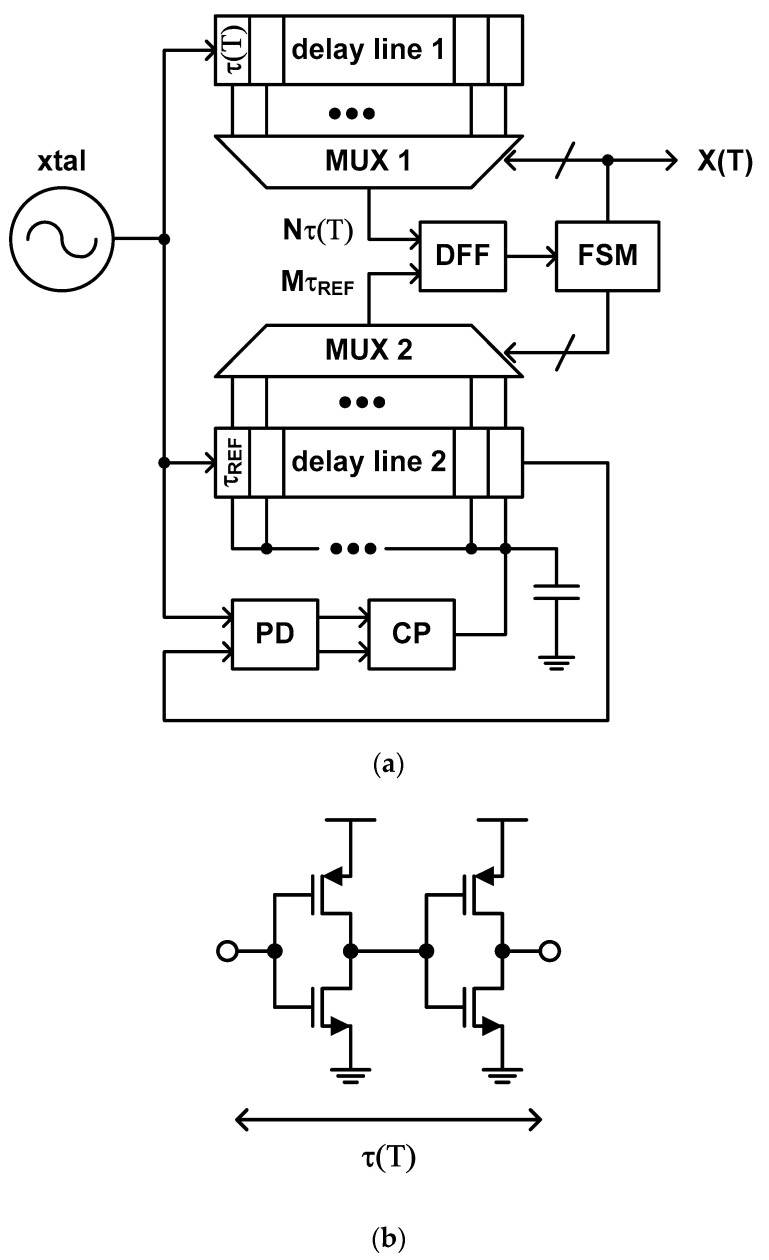
(**a**) Architecture of the type 3 time domain CMOS temperature sensor and (**b**) the inverter delay cell.

The temperature estimation function of this type of time domain CMOS temperature sensor is defined as the ratio of the temperature independent delay time and the temperature dependent delay time. As shown in Figure 5a, the temperature sensor, which was proposed by D. Ha et al. in 2012, has two delay lines, one of which generates the temperature dependent delay time and the other generates the temperature independent delay time by synchronizing its delay time to the clock period of a temperature stable crystal oscillator applied externally with the help of a delay locked loop (DLL) which consists of a phase detector (PD), a charge pump (CP) and a loop filter in addition to the second delay line [40].

Since a CMOS inverter is used as a delay cell of the delay line as shown in Figure 5b, the delay time of each delay cell may be expressed as

(1) $$\tau \left(T\right)=\frac{L}{W}\times \frac{C_{L}}{C_{\mathrm{OX}}}\times \frac{1}{\mu \left(T\right)}\times \frac{\ln\left\{3-4\frac{V_{\mathrm{TH}}\left(T\right)}{V_{\mathrm{DD}}}\right\}}{V_{\mathrm{DD}}-V_{\mathrm{TH}}\left(T\right)}$$

under the normal DC bias condition where W and L are the channel width and length, C_L_ is the load capacitance, C_OX_ is the gate oxide capacitance per unit area and V_DD_ is the power supply voltage [56,57]. From (1), the temperature estimation function can be obtained as

(2) $$X\left(T\right)=\frac{{M\tau }_{\mathrm{REF}}}{\tau \left(T\right)}$$

where M is a digital value determined during one-point calibration at a certain calibration temperature, T_C_, to let ${M\tau }_{\mathrm{REF}}=N_{C}\tau \left(T_{C}\right)$ where N_C_ is the predetermined reference digital value of the multiplexer (MUX) 1 pairing with T_C_. In this architecture, N is finally determined by the finite state machine (FSM) after the D flipflop (DFF) compares two clock signals from MUX1 and MUX2 and decides which one is faster than the other repeatedly.

To digitize the temperature estimation function, this temperature sensor utilizes many number of fine delay cells instead of a binary counter. Since this structure does not need to wait too long time for the binary counter to finish its counting operation, it can obtain high temperature resolution as well as fast conversation rate at the same time. However, it has to occupy large die area because many number of fine delay cells are required to achieve high temperature resolution. Moreover, the inevitable mismatches between the fine delay cells distributed over large die area cannot help but worsen the temperature error even if we pay careful attention to the layout. Consequently, this temperature sensor [40] shows the relatively fast conversion rate of 5 k samples/s, the relatively large temperature error of −4.0 °C to +4.0 °C over the temperature range from 0 °C to 100 °C, and the relatively large die area of 0.12 mm^2^.

If we go along with the authors that μ(T) is much more effective than V_TH_(T) in (1) so that the temperature estimation function of (2) is almost not affected by V_TH_(T), the Equation (2) can be approximated to (3) because μ(T) is generally represented as (4) with a negative fitting coefficient of α [40,58,59,60,61,62,63]. Thus, one-point calibration can be used for process variation compensation.

(3) $$X\left(T\right)={\left(\frac{T_{C}}{T}\right)}^{\alpha }\times N_{C}$$

(4) $$\mu \left(T\right)=\left(\mu T_{0}\right)\times {\left(\frac{T}{T_{0}}\right)}^{\alpha }$$

However, since (3) is still not a linear function but an exponential function with respect to T, this temperature sensor is in need of additional digital post processing block for nonlinear-to-linear mapping to minimize the temperature error. Lastly, as the temperature estimation function of (2) is a function of not only μ(T) and V_TH_(T) but also V_DD_, this temperature sensor is necessarily sensitive to the power supply variation. Thus, this temperature sensor should be implemented along with an additional integrated voltage regulator [64,65,66,67,68,69,70,71,72,73,74,75] for constant V_DD_. The measured V_DD_ sensitivity is as large as 1.6 °C/mV with no voltage regulator.

### 3.2. Type 4

**Figure 6:**
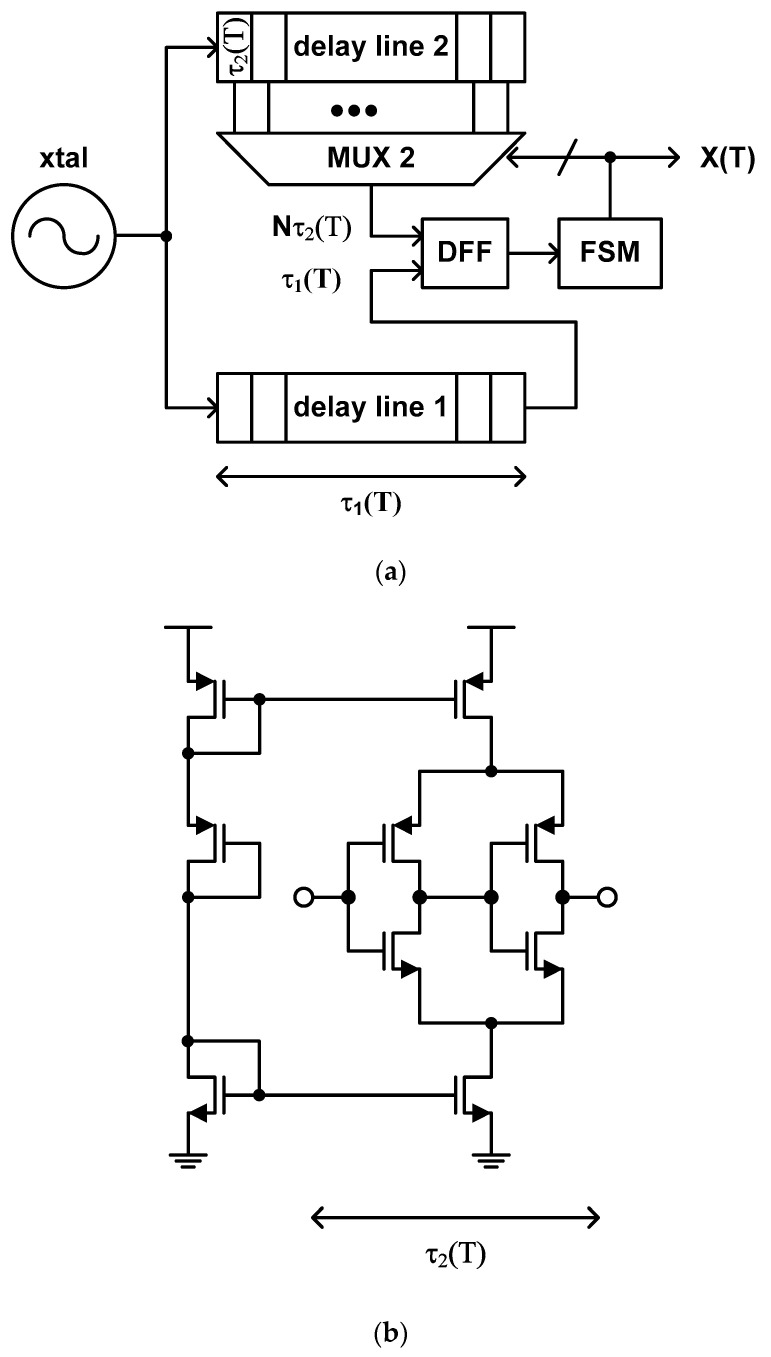
(**a**) Typical structure of the type 4 temperature sensor and (**b**) the inverter delay cell which is relatively insensitive to temperature.

The temperature estimation function of this type of time domain CMOS temperature sensor is defined as the ratio of two different temperature dependent delay times. The typical structure of this type of temperature sensor, which was proposed by P. Chen et al. in 2010 [38], includes two delay lines, of which delay times vary in a different way from each other with respect to temperature, and a successive approximation register (SAR) control logic [76,77,78,79,80,81,82,83,84,85,86,87] implemented as an FSM. For example, if one of these delay lines is composed of the general inverter type delay cells of Figure 5b, then the other is composed of the delay cells shown in Figure 6b which are less sensitive to temperature [33,38].

Thus, the temperature estimation function can be represented as

(5) $$X\left(T\right)=\frac{\tau_{1}\left(T\right)}{\tau_{2}\left(T\right)}\approx \mathrm{aT}+b$$

where a and b are the coefficients. If we accept the authors’ assertion that the order of the delay time, τ_2_(T), of the delay cell of Figure 6b with respect to temperature can be made exactly 1 less than that of the delay time, τ_1_(T), of the delay cell of Figure 5b, the temperature estimation function of (5) may become a linear equation of T and the coefficient, b, will be 0 exactly. If this is true, it is very lucky and one-point calibration may be enough for process variation compensation because we just need to determine the value of a in (5). However, since the orders of τ_1_(T) and τ_2_(T) cannot be controlled accurately, the temperature estimation function actually becomes a bit different from the linear equation of T and the coefficient, b, also cannot be zero. Thus, we should necessarily carry out two-point calibration for process variation compensation for this type of temperature sensor. The second delay line of this temperature sensor mainly functions like the digital post processing block which was used for nonlinear-to-linear mapping to reduce the temperature error of the type 3 temperature sensor.

Like the type 3, this type of temperature sensor has also a large number of delay cells instead of a binary counter to obtain a fine temperature resolution. Thus, it has fast temperature conversion rate at the cost of large die area. Of course, since it does not require a DLL anymore, we can save a small portion of active die area compared to type 3. The mismatch problem between fine delay cells distributed over large die area still exists. In [38], the implemented time domain CMOS temperature sensor shows the temperature resolution of 0.1 °C, the temperature error of −0.4 °C to +0.6 °C over the temperature range from 0 °C to 90 °C, and the very large die area of 0.6 mm^2^. Because the delay time of the CMOS inverter delay cell is a function of V_DD_ as shown in (1), this type of temperature sensor is also very sensitive to V_DD_ variation.

### 3.3. Type 5

**Figure 7:**
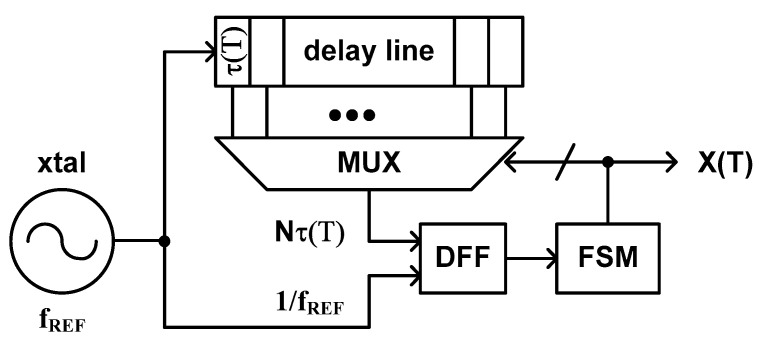
The architecture of the type 5 time domain CMOS temperature sensor.

This type of time domain CMOS temperature sensor was proposed by Z. Xu et al. in 2020 [54]. The architecture of this temperature sensor consists of a delay line, of which delay time is dependent of temperature, and a SAR control logic as shown in Figure 7. The DFF and the SAR control logic determine the number of fine delay cells, which the clock signal coming from a crystal oscillator should go through within the delay line, to make the period of the clock signal, 1/f_REF_, be equal to the delay time of the selected delay line, N × τ(T). Thus, the temperature estimation function of this type of temperature sensor is represented as follows.

(6) $$X\left(T\right)=\frac{\frac{1}{f_{\mathrm{REF}}}}{\tau \left(T\right)}\approx \mathrm{aT}+b$$

Compared to the previous type 3 and 4, this type of temperature sensor has the merit of requiring only one delay line inside. Although it still needs a large number of fine delay cells for high temperature resolution, it is true that one delay line occupies less die area than two delay lines do. This temperature sensor has fast conversion rate because it utilizes a SAR based delay line instead of a binary counter, but the temperature error is still vulnerable to the mismatch between fine delay cells distributed over large die area. The temperature error of this type of temperature sensor depends on how symmetrically and uniformly we can layout the fine delay cells and how accurately and carefully we can trim the layout of each fine delay cell through iterative post layout simulations. Additionally, since the temperature estimation function is inversely proportional to τ(T) as shown in (6), we can reduce the temperature error by adopting a specially designed delay cell which can linearize 1/τ(T) as much as possible. From (6), we can see that two-point calibration is necessary to determine the coefficients, a and b, of the temperature estimation function for process variation compensation.

The temperature sensor which was implemented in [54] shows the temperature error of −1.6 °C to +0.6 °C over the temperature range from 0 °C to 100 °C, the temperature resolution of 0.49 °C, and the relatively fast conversion rate of 25 ksamples/s. The active die area is as large as 0.432 mm^2^. Since the V_DD_ sensitivity of this temperature sensor depends on the structure of the delay cell, if the inverter type delay cell of Figure 5b is used, the temperature sensor cannot help but has poor V_DD_ sensitivity. Thus, we generally need an additional voltage regulator to keep V_DD_ as constant as possible.

### 3.4. Type 7

**Figure 8:**
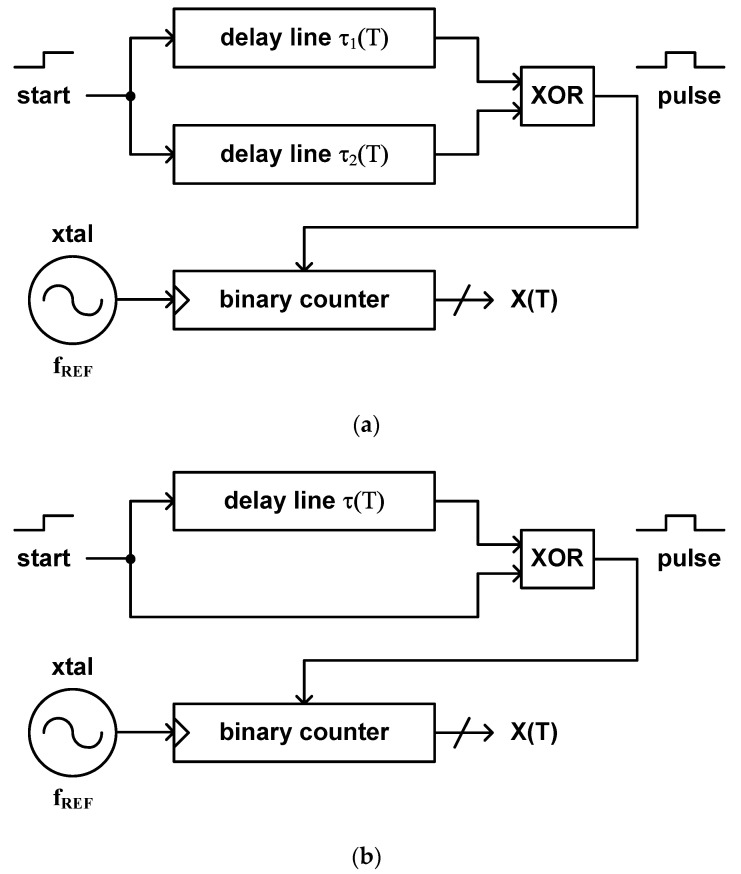
The architectures of the type 7 time domain CMOS temperature sensor containing (**a**) two delay lines and (**b**) a single delay line.

As M. K. Law et el. have proposed in 2009 and 2010, this type of time domain CMOS temperature sensor can be implemented by using two delay lines and a binary counter as shown in Figure 8a [35,37]. If two delay lines are designed to have different delay times with respect to temperature, i.e., one of which has a positive temperature coefficient and the other has a negative temperature coefficient, we can obtain a pulse signal of which pulse width equals to the difference of their delay times at the output node of the XOR gate. Then, the temperature estimation function of this type of temperature sensor is defined as the ratio of the difference between the delay times of two delay lines and the period of the temperature independent clock signal applied from an external crystal oscillator as follows.

(7) $$X\left(T\right)=\frac{\tau_{2}\left(T\right)-\tau_{1}\left(T\right)}{\frac{1}{f_{\mathrm{REF}}}}$$

If we decide to use only a single delay line to simplify the architecture, we can do it by applying the delayed start signal with the original start signal as the dual inputs of the XOR gate as shown in Figure 8b. Then, a pulse signal, of which pulse width equals to the delay time of the delay line, is generated at the output node of the XOR gate [51]. In this case, the temperature estimation function is defined as the ratio of the delay time of a single delay line and the period of the temperature independent clock signal applied from a crystal oscillator.

(8) $$X\left(T\right)=\frac{\tau \left(T\right)}{\frac{1}{f_{\mathrm{REF}}}}$$

Since this type of temperature sensor utilizes a binary counter for digitizing the temperature modulated pulse width rather than the delay line which includes a large number of fine delay cells like type 3, 4 and 5, it has a merit of occupying relatively small die area and can have a fine temperature resolution at the cost of relatively slow conversion rate. Consequently, the temperature sensor implemented in [37] has the relatively small die area of 0.0416 mm^2^, the temperature resolution of 0.14 °C to 0.21 °C and the relatively slow conversion rate of 333 samples/s. Over the temperature range from −10 °C to +30 °C, it shows the temperature error of −0.8 °C to +1.0 °C.

**Figure 9:**
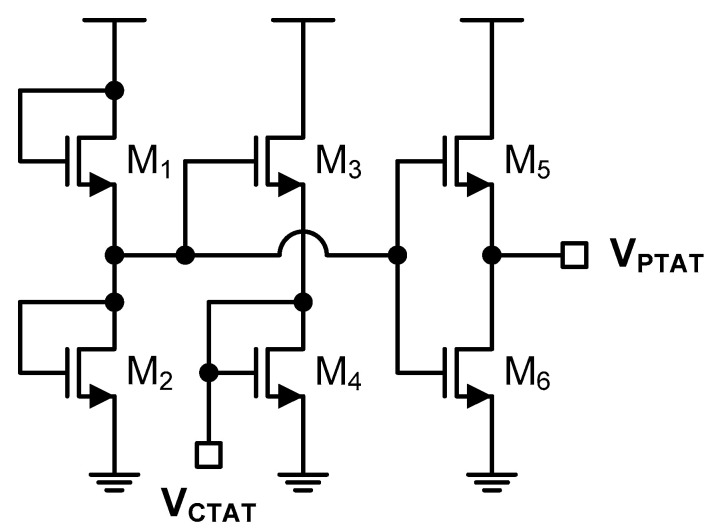
PTAT and CTAT voltage generation circuit operating in the subthreshold region.

In Figure 8, the delay lines can be implemented in various ways. One of them is to use a proportional to the absolute temperature (PTAT) and a complementary to the absolute temperature (CTAT) signals [88,89,90,91,92,93,94]. If the PTAT and CTAT voltages are generated by the NMOS transistors operating in the subthreshold region as shown in Figure 9, the PTAT and CTAT voltages can be represented as follows.

(9) $$V_{\mathrm{PTAT}}\left(T\right)=V_{T}\left[\ln\frac{{\left(\frac{W}{L}\right)}_{5}}{{\left(\frac{W}{L}\right)}_{6}}+G\left(\exp\left(-\ln\frac{{\left(\frac{W}{L}\right)}_{5}}{{\left(\frac{W}{L}\right)}_{6}}\right)\right)\right]$$

(10) $$V_{\mathrm{CTAT}}\left(T\right)=\frac{V_{\mathrm{DD}}}{4}+V_{T}\left[\frac{\ln\left(K\right)}{4}+G\left(\frac{1}{2}\exp\left(-\frac{1}{4}\left(\frac{V_{\mathrm{DD}}}{V_{T}}+\ln\left(K\right)\right)\right)\right)\right]$$

where

(11) $$K={\left(\frac{{\left(\frac{W}{L}\right)}_{3}}{{\left(\frac{W}{L}\right)}_{4}}\right)}^{2}\frac{{\left(\frac{W}{L}\right)}_{1}}{{\left(\frac{W}{L}\right)}_{2}}$$

and G(⋅) is the Lambert-W function [37]. If the PTAT and CTAT voltages are converted to the PTAT and CTAT currents like I_1_(T) = V_PTAT_(T)/R and I_2_(T) = V_CTAT_(T)/R and the delay times of the delay lines are determined as τ_1_(T) = C × V_DD_/2I_1_(T) and τ_2_(T) = C × V_DD_/2I_2_(T), respectively, as analyzed in [37], the temperature estimation function can be represented as follows.

(12) $$X\left(T\right)=\frac{\tau_{2}\left(T\right)-\tau_{1}\left(T\right)}{\frac{1}{f_{\mathrm{REF}}}}=\frac{\frac{{\mathrm{RCV}}_{\mathrm{DD}}}{2V_{\mathrm{CTAT}}\left(T\right)}-\frac{{\mathrm{RCV}}_{\mathrm{DD}}}{2V_{\mathrm{PTAT}}\left(T\right)}}{\frac{1}{f_{\mathrm{REF}}}}\approx \mathrm{aT}+b$$

Since we should determine the coefficients, a and b, of (12), for process variation compensation, two-point calibration should be carried out before temperature measurement. Lastly, if the temperature estimation function depends on V_DD_ as shown in (12), the temperature sensor may have the huge temperature error due to V_DD_ variation so that we need an additional voltage regulator to provide the constant supply voltage.

### 3.5. Type 8

**Figure 10:**
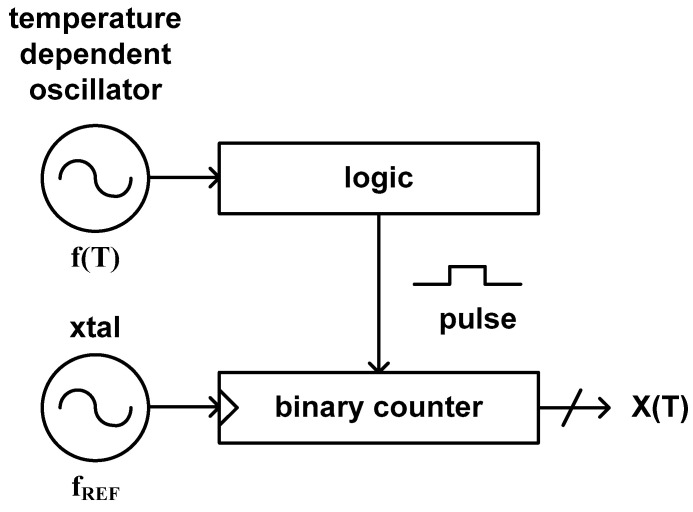
The architecture of the type 8 time domain CMOS temperature sensor.

As shown in Figure 10, this type of time domain CMOS temperature sensor consists of a temperature dependent oscillator, a digital logic generating a pulse signal of which pulse width is proportional to the clock period of the temperature dependent oscillator and a binary counter. The digital logic can be implemented by using another binary counter [43], an XNOR gate with additional clock signal [45,48] or in other ways. This type of temperature sensor estimates the temperature by counting the number of the temperature independent clock period of a crystal oscillator during the pulse width which is linear with the clock period of the temperature dependent oscillator. Thus, the temperature estimation function is defined as the ratio of the clock period of the temperature dependent oscillator and the clock period of the temperature stable crystal oscillator.

(13) $$X\left(T\right)=\frac{\frac{1}{f\left(T\right)}}{\frac{1}{f_{\mathrm{REF}}}}$$

Since this type of temperature sensor utilizes a simple digital logic instead of the delay lines of type 7 to generate the temperature modulated pulse width, it has a merit of occupying very small die area. At the same time, since it utilizes a binary counter to digitize the temperature modulated pulse width, it can achieve relatively fine temperature resolution at the price of relatively slow conversion rate. Therefore, this type of temperature sensor implemented in [43] occupies the relatively small die area of 0.07 mm^2^, the fine temperature resolution of 0.045 °C and the relatively slow conversion rate of 10 samples/s. Over the temperature range from −40 °C to +120 °C, it shows the temperature error of −1.2 °C to +0.2 °C.

If a CMOS inverter is used as a delay cell of the temperature dependent oscillator in this architecture, the delay time of the delay cell can be represented by (1) and the clock period of this oscillator will be also linear with the delay time of (1). Consequently, the temperature estimation function of (13) can be approximated to aT + b, so that we need to carry out two-point calibration for process variation compensation. Furthermore, because the delay time of (1) is dependent of V_DD_, the clock period of the temperature dependent oscillator which is composed of the CMOS inverter delay cells is also dependent of V_DD_. So we should add a voltage regulator to reduce the supply voltage variation in this case.

### 3.6. Type 11

**Figure 11:**
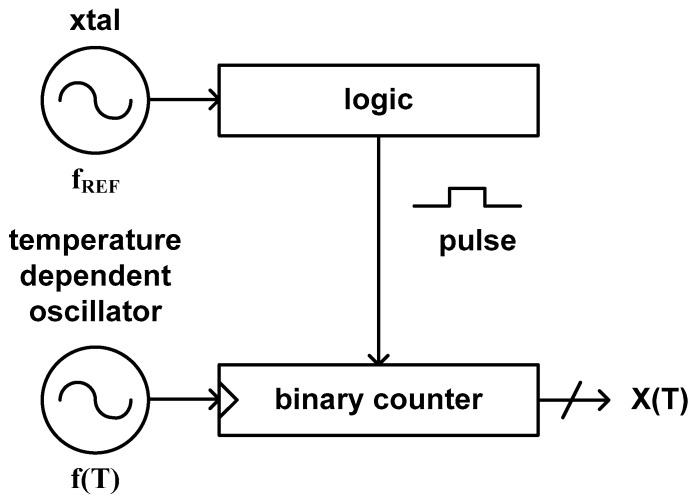
The architecture of the type 11 time domain CMOS temperature sensor.

This type of time domain CMOS temperature sensor is based on an architecture similar to but a little bit different from type 8. It is because the temperature estimation function of this type is an inverse ratio of that of type 8 as can be seen in (14). Figure 11 shows the architecture of the time domain CMOS temperature sensor published by Y.-S. Lin et al. in 2008 [34]. This architecture was also used by S. Jeong et al. and Z. Tang et al. in 2014 [46] and 2020 [55], respectively. This temperature sensor estimates temperature by counting the number of clock signals generated from the temperature dependent oscillator during the pulse width which is proportional to the temperature independent reference clock period applied from a crystal oscillator.

(14) $$X\left(T\right)=\frac{\frac{1}{f_{\mathrm{REF}}}}{\frac{1}{f\left(T\right)}}$$

Since this type of temperature sensor utilizes a digital logic, a binary counter and an integrated oscillator instead of a delay line to digitize the temperature estimation function, it can be implemented within a very small die area. However, as it counts the number of clock signals by using a binary counter, it should endure a low conversion rate to obtain a fine temperature resolution. In [46], the implemented temperature sensor occupies the relatively small die area of 0.09 mm^2^, the low conversion rate of 33 samples/s and the temperature resolution of 0.3 °C. Over the temperature range from 0 °C to 100 °C, it shows the temperature error of −1.4 °C to +1.5 °C. Specially, in this paper, for the purpose of obtaining V_DD_ insensitive temperature characteristic, an NMOS transistor operating in the subthreshold region was used as a sensing element. Because the current equation of the NMOS transistor in the subthreshold region is almost independent of V_DD_, this temperature sensor shows the relatively small temperature error of −3.15 °C to +2.5 °C against the supply voltage variation from 1.0 V to 1.4 V.

**Figure 12:**
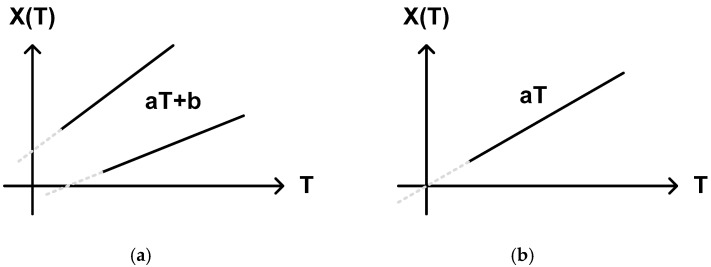
Temperature estimation functions approximated to (**a**) aT + b and (**b**) aT, respectively.

Whether we choose one-point calibration method or two-point calibration method for process variation compensation depends on the sensing element. As far as the sensing element has a good temperature linearity, the temperature estimation function can be represented by a linear function of the temperature. However, if the temperature characteristic of the adopted sensing element does not perfectly cross the zero point, the temperature estimation function may be approximated to a linear function with two coefficients like aT + b as shown in Figure 12a rather than a linear function with one coefficient like aT as shown in Figure 12b. In most of the cases, we should carry out two-point calibration to find out these coefficients, a and b.

### 3.7. Type 12

**Figure 13:**
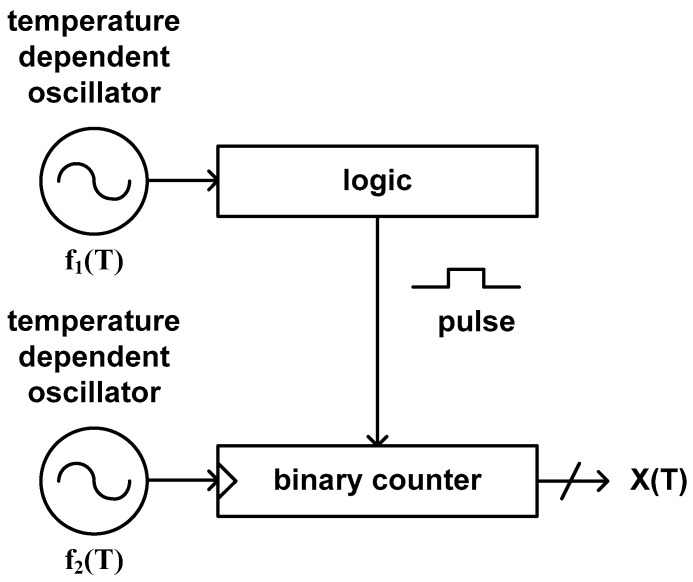
The architecture of the type 12 time domain CMOS temperature sensor.

Contrary to the previously discussed type 8 and 11, this type of temperature sensor has the temperature estimation function defined as the ratio of two different clock periods of the integrated temperature dependent oscillators. Figure 13 shows the architecture of the type 12 time domain CMOS temperature sensor implemented in [50,53]. This temperature sensor estimates temperature by counting the number of clock signals generated from the second integrated temperature dependent oscillator during the pulse width which is proportional to the clock period of the first integrated temperature dependent oscillator.

(15) $$X\left(T\right)=\frac{\frac{1}{f_{1}\left(T\right)}}{\frac{1}{f_{2}\left(T\right)}}$$

This type of temperature sensor has a few advantages compared with the previous type 8 and 11 temperature sensors. First, an integrated oscillator consumes less power than a crystal oscillator and we can even further reduce the power consumption of the integrated oscillator by utilizing a sleep mode. Second, while an off-chip crystal oscillator is physically huge, an integrated oscillator occupies less die area. So, we can reduce the form factor of the temperature sensor. Third, if we use two integrated oscillators which have similar V_DD_ sensitivities, this type of temperature sensor can be designed to be V_DD_ insensitive by canceling out the effects of V_DD_ variations from two integrated oscillators. Consequently, the implemented temperature sensor in [53] occupies the small die area of 0.074 mm^2^, the temperature resolution of 0.145 °C and the very low conversion rate of 1.2 samples/s. Over the temperature range from −20 °C to 80 °C, it shows the temperature error of −0.9 °C to 1.2 °C. At the same time, it has the V_DD_ sensitivity of as low as 3.8 °C/V when the supply voltage varies from 0.7 V to 1.5 V. For process variation compensation, this temperature sensor requires two-point calibration.

### 3.8. Other Types

In addition to the types 3, 4, 5, 7, 8, 11 and 12, there are the other types 1, 2, 6, 9 and 10 which have not yet been published in literature until now as shown in Figure 4. The type 1 has the temperature estimation function defined as the ratio of a temperature dependent delay time and a temperature independent delay time and the type 2 has the temperature estimation function defined as the ratio of a temperature dependent period and a temperature independent delay time. Meanwhile, the type 6 temperature estimation function is defined as the ratio of a temperature dependent period and a temperature dependent delay time and the type 9 temperature estimation function is defined as the ratio of a temperature independent delay time and a temperature dependent period. Lastly, the type 10 temperature estimation function is defined as the ratio of a temperature dependent delay time and a temperature dependent period.

Since the temperature estimation function of the type 1 is the reciprocal of that of the type 3 as shown in Figure 4, these two types of temperature sensors can be implemented in the similar architecture. Also, since the temperature estimation functions of the type 2 and 9 are reciprocal to each other, these types of temperature sensors may commonly consist of a DLL based delay line for generating a temperature independent delay time and an integrated oscillator for generating a temperature dependent period. Similarly, since the temperature estimation functions of the types 6 and 10 are reciprocal to each other, they may commonly consist of a delay line for generating a temperature dependent delay time and an integrated oscillator for generating a temperature dependent period. That is, the temperature sensors of the types 2, 6, 9 and 10 require both of a delay line and an oscillator at the same time which considerably complicates the architecture of the time domain CMOS temperature sensor. It seems therefore these types of temperature sensors have not yet been published in literature. Of course, if we can find some ideas to reduce the design complexity of the architecture and some appropriate applications, these types of temperature sensors may be also implemented and utilized in the near future.

## 4. Discussion

Table 1 summarizes the performances of the time domain CMOS temperature sensors referred in this paper.

**Figure 14:**
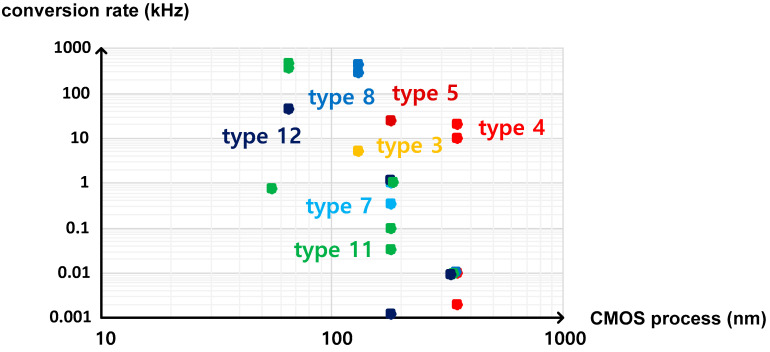
Temperature conversion rate of each type of temperature sensors versus the minimum channel length of the CMOS processes.

**Figure 15:**
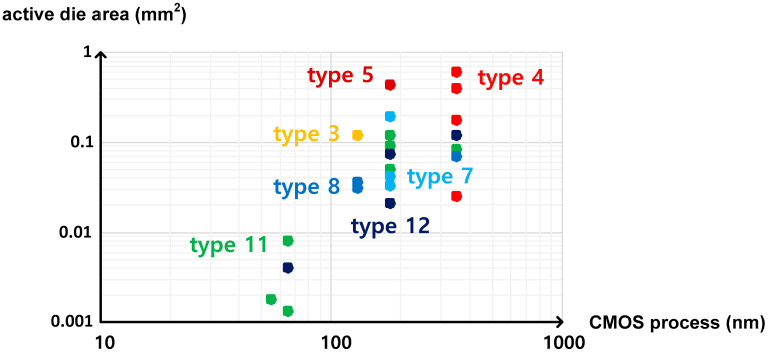
Active die area of each type of temperature sensors versus the minimum channel length of the CMOS processes.

These temperature sensors, which were categorized into twelve types on the basis of the temperature estimation function in this paper, have the distinct characteristics as follows. First, if the temperature estimation function has τ(T) or τ_REF_ in the denominator, the temperature conversion rate is relatively fast, but the active die area is relatively large. Second, if the temperature estimation function has 1/f(T) or 1/f_REF_(T) in the denominator, the temperature conversion rate is relatively slow, but the active die area is relatively small. Figure 14 and Figure 15 show the temperature conversion rate and the active die area of the time domain CMOS temperature sensors summarized in Table 1 versus the CMOS process used for implementation. As shown in the figures, the temperature sensors of type 3, 4 and 5 have the faster temperature conversion rate and the larger die area than those of type 7, 8, 11 and 12 in general if they are implemented by using the CMOS processes with the same minimum channel length.

Additionally, if the temperature estimation function has 1/f(T) at least in one of the numerator and the denominator, the temperature sensor requires additional initialization time for an integrated oscillator to stabilize its oscillation frequency. Thus, the type 8, 11 and 12 temperature sensors are not adequate for the low power temperature monitoring systems if they turn on and off the system power for energy efficiency and do not provide enough time for the initialization of the integrated oscillator. In that case, the oscillator should be turned on all times whether it is used or not.

Finally, as the temperature estimation function is approximated to aT + b in most of the cases, the process variation compensation should be done by two-point calibration. Especially only when the temperature estimation function is approximated to aT with b = 0, one-point calibration can be used. The temperature error is directly related to the temperature characteristic of a sensing element and the temperature estimation function of a temperature sensor. If the temperature estimation function has τ(T) or τ_REF_ in the denominator, the temperature error arising from the mismatch between a large number of fine delay cells distributed over large die area may be added. The supply voltage sensitivity tends to become large if a CMOS inverter type delay cell is used in the delay line or the oscillator. For obtaining low V_DD_ sensitivity, we can put τ(T) and/or 1/f(T) with almost same V_DD_ sensitivities in the numerator and the denominator of the temperature estimation function like type 4 and 12 temperature sensors to cancel out the effects of V_DD_ variations. Or, we can also make the temperature sensor less sensitive to V_DD_ variation by adopting a CMOS transistor operating in the subthreshold region because the subthreshold current of the CMOS transistor is independent of V_DD_. Otherwise, we should always implement a voltage regulator additionally to suppress V_DD_ variations in the temperature monitoring systems.

## 5. Conclusions

In this review paper, the time domain CMOS temperature sensors have been categorized into twelve types on the basis of their temperature estimation functions. The temperature estimation function is defined as the ratio of two measured time domain signals which are selected from a temperature independent delay time, a temperature dependent delay time, a temperature independent period and a temperature dependent period. The categorized temperature sensors have been discussed in terms of temperature conversion rate, die area, process variation compensation, temperature error, power supply voltage sensitivity and so on. Based on their characteristics, we can choose the most appropriate one from twelve types to satisfy a given specification.

## Figures and Tables

**Table 1:** Performance summary.

Reference	Type	CMOS Technology	Die Area	Conversion Rate	Temperature Range	Resolution	Temperature Error	V_DD_ Sensitivity
[40]	3	0.13 μm	0.12 mm^2^	5 kHz	0~100 °C	0.78 °C	−4.0~4.0 °C	1600 °C/V
[33]	4	0.35 μm	0.175 mm^2^	10 kHz	0~100 °C	0.16 °C	−0.7~0.9 °C	NA
[36]	4	0.35 μm	0.4 mm^2^	20 Hz	−40~80 °C	0.5 °C	−0.8~0.8 °C	0.12 °C/V
[38]	4	0.35 μm	0.6 mm^2^	2 Hz	0~90 °C	0.09 °C	−0.4~0.6 °C	33 °C/V
[47]	4	0.35 μm	0.025 mm^2^	10 Hz	0~100 °C	0.2 °C	−0.8~1.0 °C	NA
[54]	5	0.18 μm	0.432 mm^2^	25 kHz	0~100 °C	0.49 °C	−1.6~0.6 °C	85 °C/V
[35]	7	0.18 μm	0.0324 mm^2^	1 kHz	0~100 °C	0.3 °C	−0.8~1.0 °C	8 °C/V
[37]	7	0.18 μm	0.0416 mm^2^	333 Hz	−10~30 °C	0.21 °C	−0.8~1.0 °C	NA
[51]	7	0.18 μm	0.19 mm^2^	1 kHz	−40~85 °C	0.18 °C	−1.0~1.0 °C	NA
[43]	8	0.35 μm	0.07 mm^2^	10 Hz	−40~120 °C	0.045 °C	−1.2~0.2 °C	NA
[45]	8	0.13 μm	0.031 mm^2^	430 kHz	20~120 °C	0.595 °C	−0.63~1.04 °C	430 °C/V
[48]	8	0.13 μm	0.036 mm^2^	293 kHz	20~120 °C	0.72 °C	−2.40~2.16 °C	NA
[34]	11	0.18 μm	0.05 mm^2^	100 Hz	0~100 °C	0.3 °C	−1.6~3.0 °C	NA
[39]	11	0.35 μm	0.084 mm^2^	10 Hz	35~45 °C	0.035 °C	−0.1~0.1 °C	NA
[41]	11	65 nm	0.008 mm^2^	469 kHz	0~100 °C	0.18 °C	−1.5~1.5 °C	NA
[42]	11	65 nm	0.0013 mm^2^	366 kHz	−40~110 °C	0.34 °C	−2.9~2.7 °C	NA
[46]	11	0.18 μm	0.09 mm^2^	33 Hz	0~100 °C	0.3 °C	−1.4~1.5 °C	15.75 °C/V
[52]	11	0.18 μm	0.118 mm^2^	1 kHz	−20~120 °C	0.048 °C	−2.0~2.0 °C	NA
[55]	11	55 nm	0.00177 mm^2^	763 Hz	−40~125 °C	0.016 °C	−0.7~0.7 °C	5.76 °C/V
[44]	12	0.35 μm	0.12 mm^2^	10 Hz	0~90 °C	0.05 °C	−0.6~0.6 °C	NA
[49]	12	0.18 μm	0.021 mm^2^	1 kHz	−30~70 °C	0.15 °C	−0.7~0.6 °C	NA
[50]	12	65 nm	0.004 mm^2^	45.5 kHz	0~100 °C	0.3 °C	−0.9~0.9 °C	34 °C/V
[53]	12	0.18 μm	0.074 mm^2^	1.2 Hz	−20~80 °C	0.145 °C	−0.9~1.2 °C	3.8 °C/V
